# Extraordinarily High Brain Natriuretic Peptide (BNP) Level in a Case of Thyroid Storm

**DOI:** 10.7759/cureus.82656

**Published:** 2025-04-20

**Authors:** Hiroaki Ishii, Katsuhiko Ohashi

**Affiliations:** 1 Department of Diabetes, Endocrinology and Metabolism, Azumino Red Cross Hospital, Azumino, JPN; 2 Department of Internal Medicine, Tokyo Metropolitan Tama-Nambu Chiiki Hospital, Tama, JPN

**Keywords:** bnp, endocrine emergency, hyperthyroidism, thyroid storm, thyrotoxicity

## Abstract

Thyroid storm is an extreme manifestation of thyrotoxicosis and represents a life-threatening endocrine emergency. Here, we report a 51-year-old woman with severe thyrotoxicosis and markedly elevated brain natriuretic peptide (BNP) level of 9950 pg/mL. Cardiac evaluation revealed poor left ventricular systolic performance. While thyroid function can affect BNP level, such extreme elevation is rare in cases of thyrotoxic crisis. This case highlighted the discrepancy between the clinical course and BNP level, emphasizing the need to recognize that BNP level may not always accurately reflect cardiac function in thyroid storm.

## Introduction

Thyroid storm is a life-threatening endocrine emergency characterized by acute deterioration over a period of days or hours with a high mortality rate [1,2]. In Japan, the condition has a mortality rate exceeding 10%, with most cases arising from untreated or poorly controlled Graves’ disease [3]. Thyroid storm is a severe form of thyrotoxicosis that can lead to multiple organ failure, often triggered by severe stress. Thyrotoxicosis can also exacerbate preexisting cardiac disease, increasing the risk of atrial fibrillation and congestive heart failure.

Here, we describe a 51-year-old woman who developed thyroid storm alongside influenza A infection. She had heart failure with reduced ejection fraction (HFrEF) with markedly elevated BNP levels. This case report highlights the discrepancy between the clinical course and the BNP level.

## Case presentation

A 51-year-old woman presented to the outpatient clinic with fever and difficulty breathing. She had been healthy until her 40s when she was diagnosed with Graves’ disease and began treatment with an antithyroid agent (methimazole 30mg). However, she stopped visiting the clinic three years ago due to confusion regarding her family doctor’s suggestion of surgical treatment. Following treatment withdrawal, she often experienced physical fatigue and palpitations. The day before presentation, she developed a high fever and cough. The following day, her family brought her to our hospital due to altered consciousness. 

**Figure 1:**
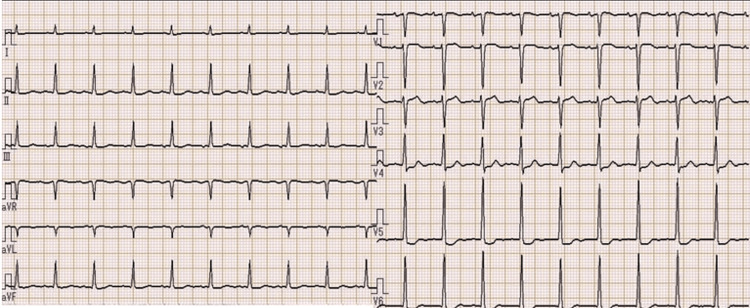
ECG on admission. ECG data: heart rate 115 bpm; PR 0.133 sec; QT 0.313 sec; RV5+SV1 6.62 mV. ECG showed sinus tachycardia, ST elevation in leads V2-3, and ST depression in leads V4-6, with high voltage in leads V5-6.

**Figure 2:**
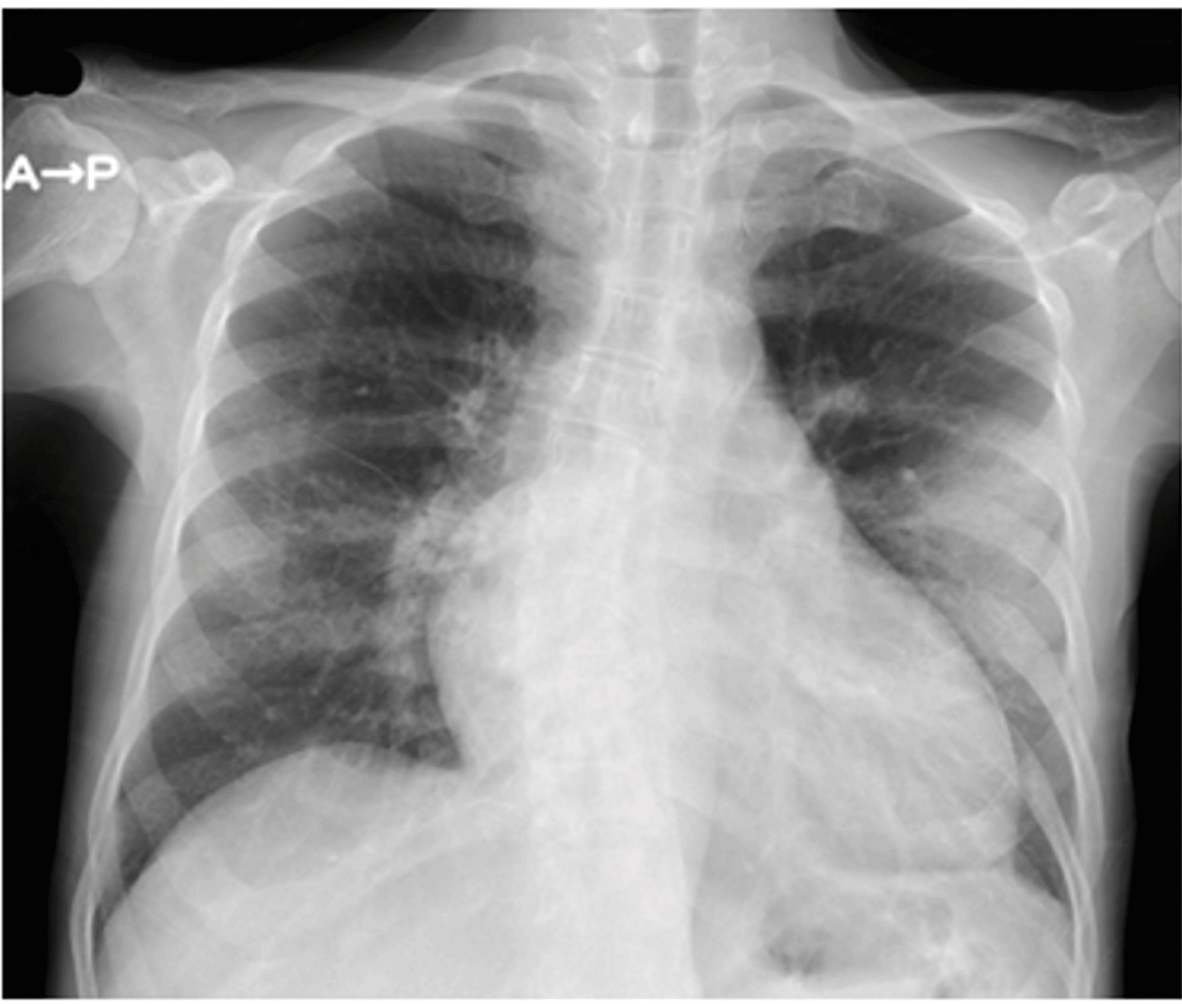
Chest X-ray on admission.

**Figure 3:**
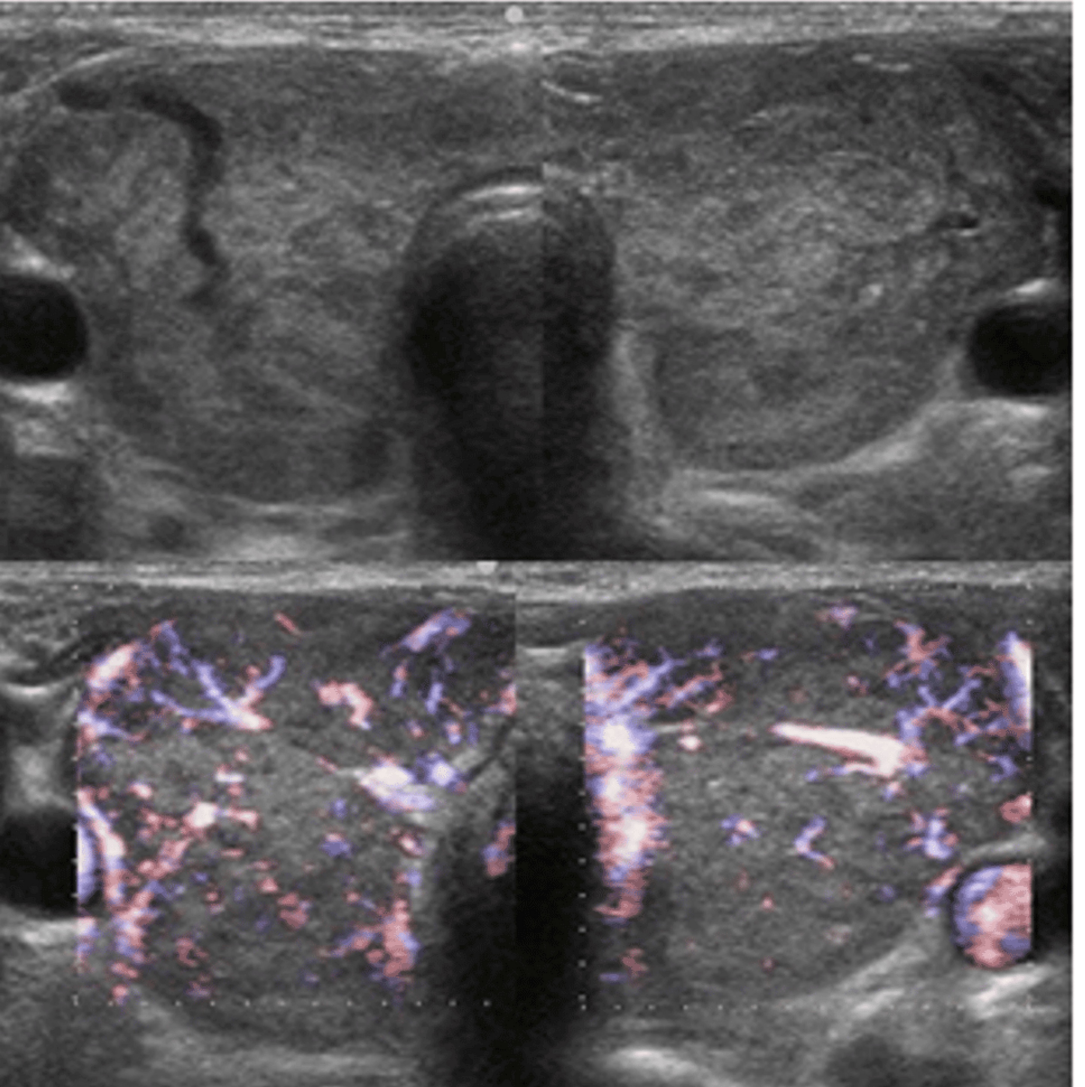
Ultrasonography on admission.

Physical findings on admission included clouding of consciousness, high-grade fever (39-40°C), tachycardia (110-140 beats per minute) with regular rhythm, finger tremor, and excessive sweating. She had diarrhea two days previously. Her thyroid gland was enlarged but showed no spontaneous pain or tenderness. Complete blood count (CBC) showed mild anemia (Hb 9.7 g/dL), and results of endocrinological tests revealed apparent hyperthyroidism and extraordinarily high BNP level (Table 1). Electrocardiogram (ECG) revealed tachycardia and ST depression in leads V4-6, with high voltage in leads V5-6 (Figure 1). Chest X-ray showed enlargement of the cardiac silhouette and irregular shadows on the left lung (Figure 2). Ultrasound showed no nodules in the thyroid gland, and blood flow was clearly increased (Figure 3). 

**Table 1:** Blood test data on admission. TRAb, thyrotropin receptor antibody; BNP, brain natriuretic peptide

Parameters	Patient values	Reference values
WBC (/µl)	3500	3500 – 9000
Hemoglobin (g/dl)	9.7	11.3 – 15.0
Platelets (×104/µl)	10.8	13.5 – 40.0
C-reactive protien (mg/dl)	0.68	0.00 – 0.30
Free T4 (ng/dl)	> 5.00	0.71 – 1.85
Free T3 (pg/ml)	> 20.00	1.92 – 3.38
Thyroid-stimulating hormone (μIU/mL)	below 0.00	0.49 – 4.67
Anti-thyroglobulin antibody(IU/mL)	1720	0.00 – 4.10
Anti-thyroid peroxidase antibody (IU/mL)	> 2000	0.00 – 5.60
TRAb (IU/L)	35.0	0.0 – 1.9
BNP	9949.6	0.0 – 18.4

The patient was admitted for emergency treatment due to suspected thyroid storm. Hydrocortisone and inorganic iodide were started concurrently with methimazole. Echocardiogram showed left ventricular enlargement and decreased ejection fraction (EF) of 40% (Table 2). We administered diuretic agents and beta-adrenergic receptor antagonists (β-AAs). Treatment was begun with peramivir and acetaminophen for influenza A virus infection and with antimicrobial agents for pneumonia. These conservative treatments improved her consciousness and achieved hemodynamic stability.

**Table 2:** Changes in echocardiogram, BNP, and thyroid function. LVDd, left ventricular end-diastolic diameter; LVDs, left ventricular end-systolic diameter; IVS, interventricular septal thickness; LVPW, left ventricular posterior wall; EF, left ventricular ejection fraction; FS, fractional shortening; LAD, left atrial dimension; E/A, early diastolic wave/atrial systolic wave; DCT, deceleration time; BNP, brain natriuretic peptide

Parameters	On admission	After one week	After two months
LVDd/Ds (mm)	51/40.8	51.8/36.7	40/26
IVS/LVPW (mm)	8.1/8.5	9.3/9.1	10.4/9.4
EF (%)/%FS	40.7/20	55.6/29.1	64/34
LAD (mm)	44	49.4	41
E/A	-	3.46	0.91
DCT (ms)	72.1	301	187
BNP (pg/mL)	9945	4859	25.9
FT4 (ng/dL)	>5.00	2.73	1.32
FT3 (pg/mL)	>20.0	2.24	5.90
TSH (μIU/mL)	below 0.00	below 0.00	below 0.00

**Figure 4:**
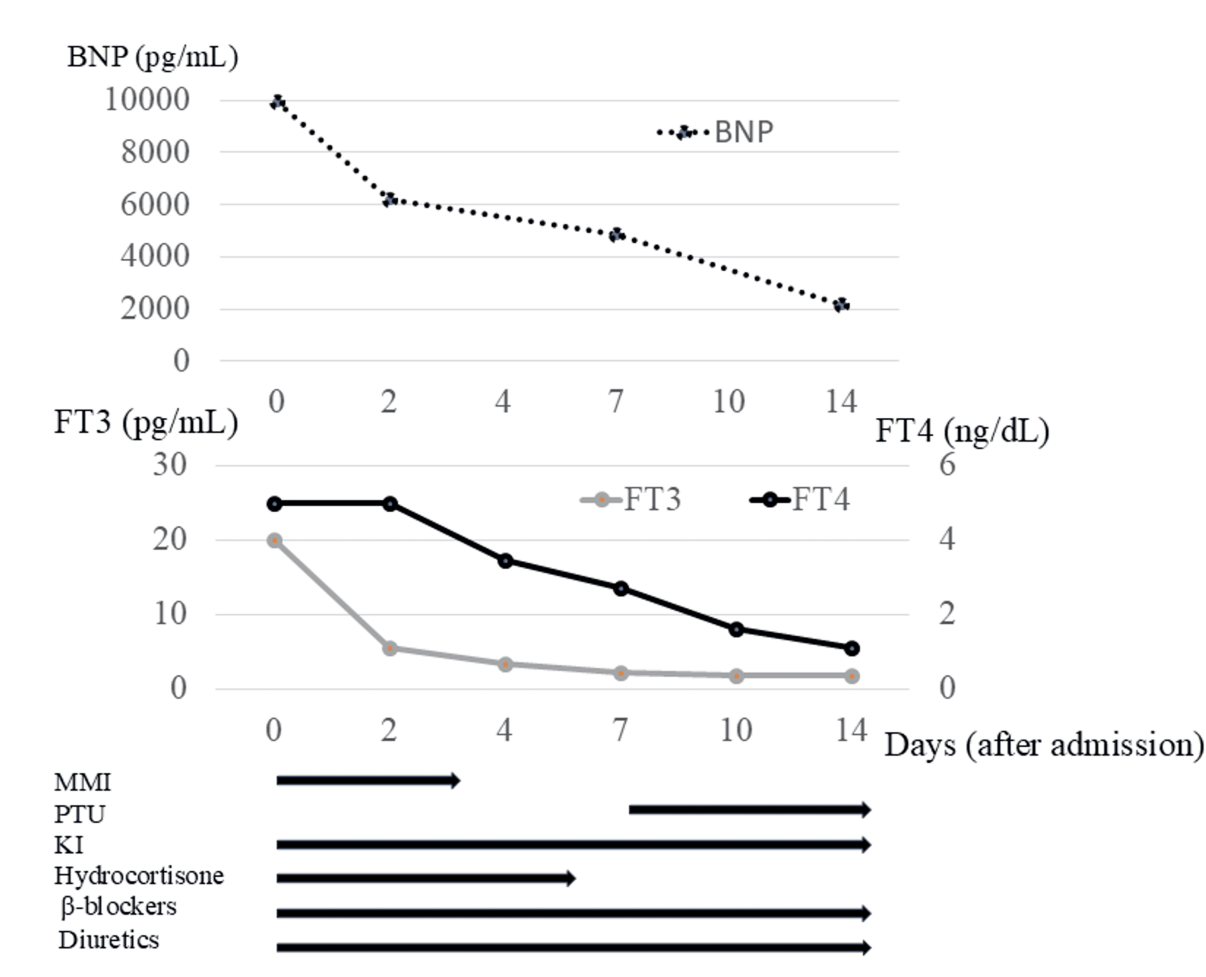
Changes in thyroid function and BNP Values of FT4 > 5.00 ng/mL and FT3 > 20.0 were not measured. FT4, free thyroxine; FT3, free triiodothyronine; BNP, brain natriuretic peptide; MMI, methimazole; PTU, propylthiouracil; KI, potassium iodide

Hyperthyroidism improved in a few days, but the BNP level was high (Figure 4). One week after admission, left ventricular diastolic failure persisted, but EF showed improvement (Table 2). On the other hand, the BNP level was still high, and thyroid function was normalized. After two weeks, her cardiac function had improved, and she did not require diuretic agents or β-AAs. The patient was discharged with no impairment in activities of daily living (ADL). After two months of admission, her diastolic dysfunction improved, and BNP showed normalization. This case showed a discrepancy between the clinical course and the BNP level.

## Discussion

Thyroid storm is a life-threatening condition often triggered by severe physical stress in patients with thyrotoxicosis. It is an endocrine emergency that leads to multiple organ failure due to the breakdown of compensatory mechanisms. One of the major symptoms is circulatory failure, manifesting as breathlessness or palpitations due to heart failure. About 40% of patients with thyroid storm also have heart failure [4]. Hyperthyroidism increases catecholamine sensitivity, leading to reduced peripheral vascular resistance, elevated heart rate, and a hyperkinetic state-changes that increase cardiac output [5]. On the other hand, peripheral tissue oxygen demand increases and leads to disruption of tissue metabolism. Myocardial damage combined with tachyarrhythmia can lead to heart failure with low output. The severity of thyrotoxicosis was remarkable in this case. Based on the Japan Thyroid Association diagnostic criteria for thyroid storm (1st edition), tachycardia and gastrointestinal symptoms, such as diarrhea, are suggestive of thyroid storm. These were major symptoms in this case. Echocardiogram showed reduced EF and mild mitral insufficiency with extraordinarily elevated BNP level.

BNP is a member of the natriuretic peptide family, which is produced in ventricular cardiomyocytes and secreted in response to volume expansion or pressure overload. Studies in patients with hyperthyroidism have shown that an increase in thyroid hormone levels results in reduced peripheral vascular resistance, elevated heart rate, and increased cardiac output [5]. Thyroid hormone promotes the secretion of BNP, stimulated by the stretching of atrial myocardial tissue. Previous studies showed that free T3 hormone directly stimulates the secretion of BNP from myocardial cells by increasing its gene expression [6-8]. In this case, thyrotoxicosis would have influenced the elevation of BNP, but the reason for the extraordinarily high level is not known. There were no findings of acute myocarditis or pericarditis associated with virus infection. Kishida et al. reported that BNP levels remained elevated despite compensation of heart failure and well-controlled heart rate as long as hyperthyroidism persisted [9]. This case showed a high BNP level despite improved thyroid function.

BNP and NT-proBNP are useful as biomarkers for heart failure (HF); they are key parameters to evaluate the severity of HF [10,11]. Patients with a BNP level of 200 pg/ml in heart failure should be started on treatment; however, this case showed 9950 pg/ml surprisingly. In this case, the echocardiogram showed improving EF for only one week from admission, and she was diagnosed with heart failure with preserved EF (HFpEF). HFpEF in hyperthyroidism is about 3%, but the BNP level with HFpEF in hyperthyroidism is unclear. About 30% of HFpEF in general (not hyperthyroidism) is normal range for BNP level [12], but this case showed a high level of BNP. We think that the BNP level might be high in HFpEF with hyperthyroidism compared to the general population.

In this case, thyrotoxicosis improved in a few days after admission, and cardiac function improved within a week, but normalization of BNP level needed two months. Kato et al. reported that the cardiovascular condition associated with thyrotoxicosis is a major factor underlying increased BNP level, but thyrotoxicosis itself contributes only to a limited extent [13]. The mortality rate of severe heart failure with thyrotoxicosis is about 10-40% [3], but most cases show improvement of cardiac function [14]. Early hemodynamic stability using antithyroid drugs and β-AAs is important for improving prognosis [15].

## Conclusions

This case showed that there may be discrepancies between actual cardiac function and BNP levels in cases of thyroid storm. We should keep in mind that there is a risk of overestimating the BNP level in thyrotoxicosis. Checking the echocardiogram each time and evaluating actual cardiac function would be critical to assess hemodynamics in thyroid storm.

## References

[1] Gavin LA (1991). Thyroid crises. Med Clin North Am.

[2] Tietgens ST, Leinung MC (1995). Thyroid storm. Med Clin North Am.

[3] Akamizu T, Satoh T, Isozaki O (2012). Diagnostic criteria, clinical features, and incidence of thyroid storm based on nationwide surveys. Thyroid.

[4] Isozaki O, Satoh T, Wakino S (2016). Treatment and management of thyroid storm: analysis of the nationwide surveys: the taskforce committee of the Japan Thyroid Association and Japan Endocrine Society for the establishment of diagnostic criteria and nationwide surveys for thyroid storm. Clin Endocrinol (Oxf).

[5] Osuna PM, Udovcic M, Sharma MD (2017). Hyperthyroidism and the heart. Methodist Debakey Cardiovasc J.

[6] Kohno M, Horio T, Yasunari K (1993). Stimulation of brain natriuretic peptide release from the heart by thyroid hormone. Metabolism.

[7] Liang F, Webb P, Marimuthu A, Zhang S, Gardner DG (2003). Triiodothyronine increases brain natriuretic peptide (BNP) gene transcription and amplifies endothelin-dependent BNP gene transcription and hypertrophy in neonatal rat ventricular myocytes. J Biol Chem.

[8] Schultz M, Faber J, Kistorp C, Jarløv A, Pedersen F, Wiinberg N, Hildebrandt P (2004). N-terminal-pro-B-type natriuretic peptide (NT-pro-BNP) in different thyroid function states. Clin Endocrinol (Oxf).

[9] Kishida C, Naito R, Kasuya H (2018). Heart failure with hyperthyroidism demonstrating discrepancy between the clinical course and B-type natriuretic peptide levels. Intern Med.

[10] Nishikimi T, Nakagawa Y (2021). Potential pitfalls when interpreting plasma BNP levels in heart failure practice. J Cardiol.

[11] Don-Wauchope AC, McKelvie RS (2015). Evidence based application of BNP/NT-proBNP testing in heart failure. Clin Biochem.

[12] Verbrugge FH, Omote K, Reddy YN, Sorimachi H, Obokata M, Borlaug BA (2022). Heart failure with preserved ejection fraction in patients with normal natriuretic peptide levels is associated with increased morbidity and mortality. Eur Heart J.

[13] Kato K, Murakami H, Isozaki O, Tsushima T, Takano K (2009). Serum concentrations of BNP and ANP in patients with thyrotoxicosis. Endocr J.

[14] Goldman LE, Sahlas DJ, Sami M (1999). A case of thyrotoxicosis and reversible systolic cardiac dysfunction. Can J Cardiol.

[15] Wada T, Sawamura A, Sugano M (2010). A report of four cases diagnosed to have thyroid crisis [Article in Japanese]. J Jpn Soc Intensive Care Med.

